# FIB-SEM as a Volume Electron Microscopy Approach to Study Cellular Architectures in SARS-CoV-2 and Other Viral Infections: A Practical Primer for a Virologist

**DOI:** 10.3390/v13040611

**Published:** 2021-04-02

**Authors:** Valentina Baena, Ryan Conrad, Patrick Friday, Ella Fitzgerald, Taeeun Kim, John Bernbaum, Heather Berensmann, Adam Harned, Kunio Nagashima, Kedar Narayan

**Affiliations:** 1 Center for Molecular Microscopy, Center for Cancer Research, National Cancer Institute, National Institutes of Health, Bethesda, MD 20892, USA; valentina.baenaecheverri@nih.gov (V.B.); ryan.conrad@nih.gov (R.C.); patrick.friday@nih.gov (P.F.); ella.fitzgerald@nih.gov (E.F.); taeeun.kim@nih.gov (T.K.); heather.berensmann@nih.gov (H.B.); adam.harned@nih.gov (A.H.); nagashimak@mail.nih.gov (K.N.); 2 Cancer Research Technology Program, Frederick National Laboratory for Cancer Research, Frederick, MD 21701, USA; 3 National Institute of Allergy and Infectious Diseases, Division of Clinical Research, Integrated Research Facility at Fort Detrick (IRF-Frederick), Frederick, MD 21702, USA; bernbaumjg@niaid.nih.gov

**Keywords:** FIB-SEM, volumeEM, vEM, SARS-CoV-2, virological synapse, segmentation

## Abstract

The visualization of cellular ultrastructure over a wide range of volumes is becoming possible by increasingly powerful techniques grouped under the rubric “volume electron microscopy” or volume EM (vEM). Focused ion beam scanning electron microscopy (FIB-SEM) occupies a “Goldilocks zone” in vEM: iterative and automated cycles of milling and imaging allow the interrogation of microns-thick specimens in 3-D at resolutions of tens of nanometers or less. This bestows on FIB-SEM the unique ability to aid the accurate and precise study of architectures of virus-cell interactions. Here we give the virologist or cell biologist a primer on FIB-SEM imaging in the context of vEM and discuss practical aspects of a room temperature FIB-SEM experiment. In an in vitro study of SARS-CoV-2 infection, we show that accurate quantitation of viral densities and surface curvatures enabled by FIB-SEM imaging reveals SARS-CoV-2 viruses preferentially located at areas of plasma membrane that have positive mean curvatures.

## 1. Introduction

In the study of virus-cell interactions, high-resolution images that provide cellular context and ultrastructural information can provide unique and important insights complementary to those acquired by light microscopy. Electrons can yield information at nanometer and sub-nanometer resolutions, allowing the observation of minute architectural details of cells and tissues. Electron microscopy (EM) has a long tradition in cell biology [1] and has played an indispensable role in the characterization of viral particles [2,3]. More recently, advances in cryo EM are providing insights into cell biology and viruses at high-resolutions and with 3-D information [4,5,6]. In parallel, several newly developed room temperature EM methods together being called volume electron microscopy or volume EM (vEM) [7] can now interrogate significantly larger sample volumes at nanometer-level resolutions. Of these vEM techniques, FIB-SEM has the resolving power in x, y, and z axes to accurately map and reconstruct features such as <100 nm-sized viral particles in all three dimensions, while also being able to probe whole mammalian cell volumes. This makes FIB-SEM a well-suited approach to study virus-cell and cell-cell interactions in high resolutions and 3-D. The current coronavirus disease 2019 (COVID-19) pandemic has triggered spectacular world-wide scientific efforts to characterize its causative agent, the betacoronavirus severe acute respiratory syndrome coronavirus 2 (SARS-CoV-2) [8]. In addition to genetic, transcriptomic, molecular and structural investigations, important details in viral pathogenesis including viral exit, entry, and cell-cell transfer can be illuminated by high-resolution 3-D analyses of virus-host cell interactions by vEM techniques such as FIB-SEM. In this primer, we emphasize practical aspects of a room temperature FIB-SEM experiment for a virologist or cell biologist looking to utilize this technology, using the example of SARS-CoV-2 infection in vitro. We also share intriguing observations made in this system to highlight possibilities enabled by FIB-SEM imaging of virus infected cells.

## 2. Electron Microscopy of Virus-Cell Interactions

“Conventional”, i.e., thin-section imaging of heavy-metal stained, resin-embedded samples by transmission electron microscopy (TEM) has helped characterize viral and cellular ultrastructure at unparalleled resolutions, including emergent viruses that may pose immediate threats to human and animal populations [9,10,11,12]. Yet, a 2-D electron micrograph, no matter how high the resolution, can only provide an incomplete picture of these 3-D processes. Room temperature electron tomography (ET) enables the extraction of 3-D information from resin sections (about half a micron thick, usually less) by computationally combining images acquired at increasing tilt angles into a tomogram [13,14]. ET has helped characterize viral replication organelles induced by SARS-CoV-2 [15,16] and cryo ET has been used to describe RNA association with and egress from intracellular double membrane vesicles, which are part of the viral replication organelles within the infected cell [17,18]. Indeed, the focused ion beam is increasingly deployed at cryogenic temperatures to thin down cellular samples for cryo ET [19,20], but here we restrict our discussions to room temperature experiments. Undoubtedly, ET has been a powerful tool to study virus-cell interactions [21,22,23], for reviews see [6,24,25], yet it is somewhat limited when whole cell or tissue-level 3-D information is required. Tomograms of sequential sections are possible [26,27,28] but are technically difficult and are plagued by beam-and knife-induced distortions at section junctions. Below, we introduce emerging EM technologies better equipped for efficiently investigating large volumes in three dimensions while retaining the ability to resolve viral particles.

## 3. Volume Electron Microscopy

**Figure 1:**
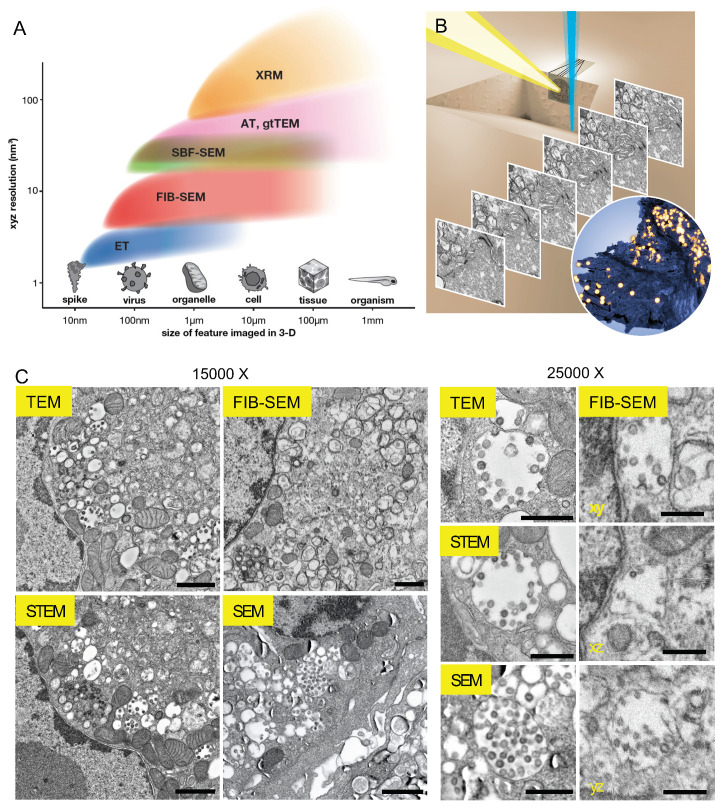
Focused ion beam scanning electron microscopy (FIB-SEM) versus other electron microscopy (EM) approaches in imaging viruses and cells. (**A**) FIB-SEM occupies a crucial gap in a plot of typical resolutions achieved against the size of volumes imaged by volume EM (vEM) techniques. XRM: X-ray microscopy, AT: array tomography, gtTEM: grid-tape TEM, SBF-SEM: serial block face SEM, FIB-SEM: focused ion beam SEM, ET: electron tomography. (**B**) Schematic of FIB-SEM acquisition. Iterative gallium ion milling (blue beam) and scanning electron imaging (yellow beam) produce serial micrographs, which are aligned and segmented to reveal features such as infected cell membranes (dark blue) and viral particles (yellow spheres). (**C**) Representative images of SARS-CoV-2 infected Vero E6 cells taken by different EM imaging modalities. Images were acquired at ~15,000× (left) or ~25,000× (right) magnifications. TEM: transmission electron microscopy, STEM: scanning transmission electron microscopy, FIB-SEM xy, xz, yz: representative slices from orthogonal planes of the reconstructed volume. The raw FIB-SEM and SEM images were contrast inverted to match TEM and STEM micrographs visually. Scale bars: 1 µm (left), 500 nm (right).

A powerful advance in EM has been the extension of imaging deep into the third (z) dimension. Electrons have a relatively short penetration distance; tens of nanometers or less into stained, resin-embedded biological samples at the lower accelerating voltages and currents typically used in a scanning electron microscopy (SEM) imaging experiment [29]. Therefore, large-scale volumetric imaging in EM is achieved by the sequential imaging of consecutive sections cut from a sample; this generates a stack of 2-D EM images which can be computationally aligned and reconstructed into a 3-D image containing information from the entire volume. Recently developed vEM methods achieve this in various ways; most prioritize automation and computation with the aim of large-scale volume acquisition. An approximate but informative way to gauge the utility of various vEM approaches is by plotting the volume of a specimen imaged in a single acquisition run against the nominal best resolution of the resulting 3-D reconstruction (Figure 1A). ET is appropriate for reconstructing smaller features at well under 10 nm resolutions, but each tomogram represents a volume no more than several hundreds of nanometers thick. Very large volumes can be interrogated by TEM-based techniques: the traditional approach of serial-section TEM requires manual collection of consecutive thin (~50 nm) sections of stained, resin-embedded specimens which are then individually imaged [30,31], while GridTape TEM uses a tape-reel to help automate section collection and imaging [32]. Newer plasma focused ion beam (pFIB) or broad ion beam (BIB) [33,34] milling and X-ray microscopy (XRM) [35] approaches are being developed, but to our knowledge have not been shown to resolve viral particles in resin-embedded cell samples. Combinations of these approaches may smartly couple sparse sampling of a large volume with high-resolution imaging of a smaller region-of-interest (ROI) [35,36], but for brevity we do not discuss this here.

In the past decade, a range of methods that provide 3-D information using SEM imaging have been deployed successfully in cell biology [7,37]. Fueled by the ability of modern SEMs to probe wide areas, vEM imaging of large volumes by SEM is now possible [38]. Conventional SEM of dried samples detects secondary electrons (SEs) arising from the inelastic interactions between incident electrons and the atoms at or close to the sample surface in a topography-sensitive manner; these give the perception of depth in stunning SEM micrographs that in reality are 2-D images. TEM-like projection images can be acquired with an SEM equipped with scanning transmission electron microscopy (STEM) instrumentation, where the electron beam is focused and rastered over a thin sample held on a TEM grid [39]. Most commonly in biological experiments, the sample is stained and resin-embedded material sectioned at a microtome, so STEM can be used for high-resolution imaging of thin sections as well as for tomography of slightly thicker samples [40,41]. STEM has been successfully used for tomographic imaging of up to 1.5 μm-thick sections of biological samples [42,43], including virus-infected cells [44], though its potential for high-throughput vEM remains to be evaluated.

Back-scatter electrons (BSEs), generated from near-elastic interactions between the incident beam and high-atomic number or “heavy” elements in the specimen, generate typically the most useful signal for SEM-based vEM. Contrast in the BSE signal arises from heavy metal stains (such as osmium, uranium salts) central to conventional TEM sample preparation protocols, and BSE derived signals dominate if topological features are minimized. Therefore, if a heavy metal stained, resin embedded sample can be planed or sectioned, the newly revealed flat surface can be efficiently imaged by the SEM to yield a TEM-like image of the surface, with inverted contrast, recorded at a BSE detector. By trading larger and uninterrupted image-able areas (no TEM grid bars) for slightly lower spatial resolutions at higher magnifications, automated iterations of sectioning and imaging cycles can in theory cover mm-size volumes with few manual interventions; this in essence is the idea behind vEM approaches.

We mention two vEM techniques that combine manual sectioning with SEM imaging. In array tomography (AT), analogous to GridTape TEM, a device called the Automated Tape-Collecting Ultramicrotome (ATUM) [45] couples a tape reeling device to a microtome to collect sections sequentially, like beads on a string. The tape with the dried sections is then cut, affixed to a 4” wafer, carbon-coated, and now any section on the tape can be imaged by SEM in an automated manner, even repeatedly at desired resolutions [46]. Single-beam AT was used to generate a ~80,000 μm^3^ region of mouse neocortex at a 3 × 3 × 30 nm voxel resolution [47]. Such sections are beginning to be probed by multiple choreographed SEM beams [48], turbocharging image generation. AT is amenable to immunogold staining [49] and correlative imaging [50] but likely requires troubleshooting before use. On the other hand, serial block face SEM (SBF-SEM) bypasses the need for collecting sections by placing a moving ultramicrotome blade inside the SEM chamber [51]. The blade repeatedly scrapes away the top ~30 nm of a resin-embedded sample mounted on a pin, and the electron beam sequentially images the freshly exposed face. Large volumes at 3 × 3 × 30 nm voxel sampling have been generated in neuronal [52,53] and other cells [54,55]. An SBF-SEM set up is relatively easy to run, yet it has some limits on imageable areas and cumulative beam-induced damage; the latter can be neutralized to an extent [56]. At these resolutions both AT and SBF-SEM can resolve ~100 nm viruses, but we warn the virologist that automatically locating small particles such as viruses in massive image volumes is still an unsolved challenge, and the anisotropic nature of reconstructions can lower confidence in potential candidates. We revisit this important point later in the paper. Further, both AT and SBF-SEM, like GridTape and serial section TEM, suffer from the limitations of physical sectioning of the resin-embedded sample by a blade: the section thickness limits z resolutions, although this may be circumvented [29], and it is currently impossible to precisely generate every section at exactly the set slice thickness. There are also knife-induced artifacts that could occur either regularly or sporadically with these approaches that the virologist should consider critically.

## 4. FIB-SEM of Virus-Cell Interactions

An entirely independent approach to interrogate recessed areas in a sample is to eschew physical sectioning and instead mill the ROI with a focused ion beam (FIB) (Figure 1B). FIBs are commonly used in materials sciences, where their high sputtering efficiency is exploited to mill away materials resistant to mechanical sectioning. Gallium (Ga) is easy to ionize and handle, and the resulting Ga^+^ FIBs are stable, relatively long lived, and responsive to fine control with the use of electronic lenses. These advantages have made Ga the default ion species for biological applications, and we point the reader to reviews that describe the use of FIBs and FIB-SEM imaging in biology [37,57,58]. Here we focus on practical aspects to consider before use of this technology to address the question at hand. Investigating virus-cell interactions poses some unique challenges: the resolution must be high enough and isotropic (i.e., equal pixel size in x, y, z) to allow for accurate visualization of virus (~100 nm) and fine alterations in host cell membrane topology and internal architecture. Simultaneously, the field-of-view must be large enough to capture sparsely distributed particles or rare structures, and also to provide relevant cellular or tissue contexts across significant distances. FIB-SEM has indeed been used to detect viral particles in the context of cells and fine membranous structures such as filopodia, which viruses are known to use in order to transit from one cell to another [59,60,61]. FIB-SEM has also provided essential information on HIV-1 trafficking through surface-connected tubules [62] and cell-to-cell spreading via so-called virological synapses [63,64]. If executed correctly, FIB-SEM imaging can provide excellent 3-D resolutions over thousands of cubic microns, making it uniquely capable to address questions that require the volumetric ultrastructure of cells and viruses.

### 4.1. Instrumentation

The addition of a second (FIB) column at an angle to the electron column in an SEM allows milling and imaging on the same sample, however, constraints in design necessitate the use of a dedicated tool. In a room temperature biological experiment, a sample is secured onto a stub and coated with conductive material; the sample is milled appropriately to reveal the ROI on a flat plane, with minimal topological contributions in the subsequent BSE image. Below we introduce the three main FIB-SEM instrument designs. Note that we do not discuss cryoFIB operations here [65].

1. FIB milling is orthogonal to the top surface, SEM imaging is at a ~54° angle, and the sample is retained at the “coincidence point”, where both beams converge in focus (Figure 1B). In Zeiss instruments with ATLAS (Fibics Inc., Ottawa, ON, Canada), a patterned platinum and carbon pad is deposited over the area of interest by the FIB. Milling the pad (and resin underneath) reveals the patterned notches in cross-section along with the sample. When captured in the image, notch locations allow the SEM to correct for sample drifts in x and y, and, critically, the FIB to correct for drifts in z “on the fly”. This feedback loop allows stable and continuous milling at z slice thicknesses reliably measured up to 3 nm [66], but this geometry causes some attenuation of signal from the deepest regions of the milled trench.
2. In another approach (Thermo Fisher/FEI), the stage tilts and rises after each FIB mill for a high-resolution SEM raster, and then lowers and tilts back for the next mill, which again is orthogonal to the top surface and parallel to the “cliff face”. Markers on the top surface and cliff face adjacent to the ROI allow for drift correction. The stage can recover its location and tilt accurately, leading to small additions to total run time, and downstream alignment algorithms can correct for drifts in the imaging plane. Z thicknesses are more difficult to accurately measure, but SEM imaging is less affected by the geometry of the trench. The stage movements may also place some limits on the size and orientations of the sample allowed in the tool.
3. An “L” shaped configuration (Hitachi) has an orthogonal orientation between the FIB and SEM columns, but it is the hybrid “Feiss” (FEI Magnum FIB + Zeiss Gemini SEM) custom tool from the Hess group [38] that has shown spectacular use in biology. Here, pre-trimmed resin samples are milled at finely controlled increments and imaged at optimal parameters, and innovative Ga tip regeneration protocols vastly increase volumes that can be imaged continuously. This orientation reveals the entire milled face of the sample for imaging, but ROI location may be limited as sputtering efficiencies at the FIB currents typically used will fall off within tens or a few hundred microns at best from the sample edge.

**Instrumentation in our experiments:** We used a Zeiss Xbeam 550 FIB-SEM and acquisition software was ATLAS3D (Fibics Inc., Ottawa, ON, Canada). A movie of our workflow in the context of a CLEM/FIB-SEM experiment may be viewed at: https://www.youtube.com/watch?v=zqW6pXaU4Go, accessed on 31 March 2021.

### 4.2. Sample Preparation

Fortuitously, for room temperature FIB-SEM, sample preparation protocols for conventional TEM are largely applicable—similar fixation, heavy metal stain, dehydration and resin embedding protocols yield samples ready for FIB-SEM imaging. High-pressure freezing and freeze-substitution protocols can also be adapted [67,68,69]. An initial FIB-SEM trial on an existing resin sample, if available, is suggested, and a TEM/STEM quality control step will minimize wasted FIB-SEM instrument time. Important points to note are:

1. There is no opportunity to post-stain samples. While lead citrate or phosphotungstic acid “post-staining” of sections on TEM grids boosts signal and contrast, this is not possible in FIB-SEM experiment. This is because these protocols are executed on accessible sections, while here, the FIB continuously and automatically ablates away material in the vacuum of an SEM chamber during a run. Instead, increased en-bloc heavy metal staining of samples to boost signal is possible, such as the rOTO protocol and its variants [70].
2. One cannot perform traditional post-embedding or Tokayasu method-based [71] immunostaining during a FIB-SEM run, for the same reason as above. Modifying these immunostaining protocols so as to complete all steps before final resin-embedding is possible [72], but 5–15 nm gold particles must be enhanced to be reliably discerned, and in our hands, this can result in significant background signals.
3. Resin hardness is important for stability under the electron and ion beams. A variety of resins including lower viscosity formulations like LR White have applications in immunocytochemistry. We and others have observed [72] that softer and even some medium hardness resin formulations perform poorly under the abrasive FIB and the constant switching between positively charged Ga ions and negatively charged electrons during a run. Hard formulations are safe and Durcupan is popular especially for tissue work [38], with newer formulations being developed.
4. Sample orientation and trimming is critical for FIB-SEM. As milling efficiency typically drops off within 100 µm, it is important to position the ROI at or just below the top surface, and firmly fix this to the stub to prevent sample movements. Adherent cells are relatively easy, as they are typically milled “upside down” following resin embedment, substrate (gridded cover slip for correlative experiments) removal, and ROI location [66]. Targeted ROI imaging in tissue samples requires correlation, which can be done with near infrared branding [73], but this still requires careful trimming before FIB-SEM steps. For deeply embedded features that do not require exact spatial correlation, aggressive semi-thick microtome sectioning and occasional visual check by toluidine blue staining is commonly used to approach or even expose the feature of interest.

**Figure 2:**
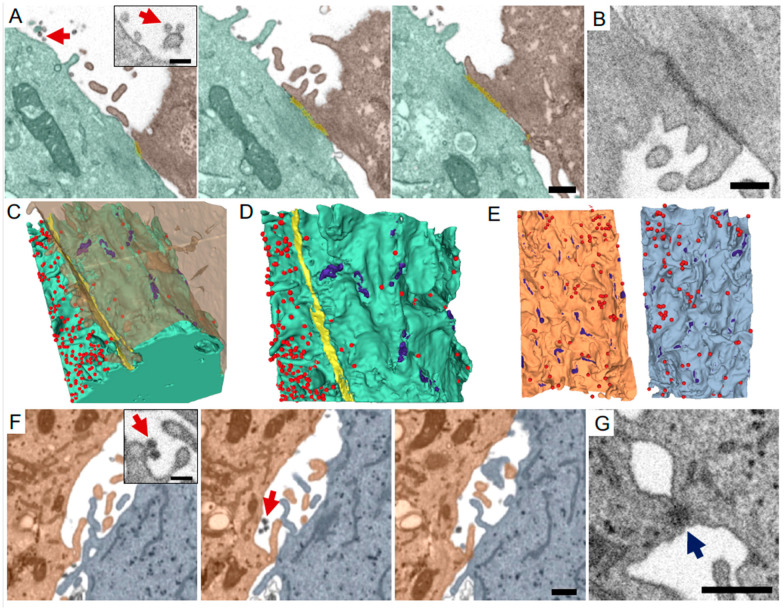
FIB-SEM imaging and 3-D reconstructions of SARS-CoV-2 viruses and cell-cell contacts. Infected Vero E6 cells (**A**–**D**) or Calu-3 human lung epithelial cells (Cortese et al., 2020) (**E**–**G**) processed for and imaged by FIB-SEM. (**A**) Periodic FIB-SEM micrographs of every 10th 8 nm slice showing a tight junction-mediated contact (yellow) between two Vero E6 cells (green and brown) and viruses in the extracellular space (red arrows). Inset shows high magnification view of viruses and cell membranes. (**B**) Higher magnification micrograph of a tight junction connecting the two cells in A. (**C**,**D**) 3-D reconstructions of plasma membranes of both cells in A (**C**). Virus particles appear as red spheres, small focal adhesions (likely desmosomes) are shown in purple, tight junction is shown in yellow. Viral density is dramatically different on either side of the contiguous tight junction (**D**). (**E**) 3-D reconstructions of two neighboring lung epithelial cells in contact (orange, light blue), separated to reveal the surfaces in closest proximity. Desmosome-like junctions were seen between the two cells (purple, and **G**). Viral particles (red) are evenly distributed on the plasma membranes. (**F**) FIB-SEM micrographs of every 5th 8 nm slice from lung epithelial cells [15]. Same cells as in E. Red arrows mark viruses. (**G**) Higher magnification micrograph of a desmosome (blue arrow) between the two cells in E-F. Scale bars (**A**,**F**,**G**): 500 nm. (**A**,**F**) insets: 200 nm. (**B**): 300 nm.

**Sample preparation in our experiments:** Sample preparation involving infectious SARS-CoV-2 was done at IRF following institutional guidelines. Vero E6 cells were seeded in T75 flasks at 80% confluency and infected with SARS-CoV-2 USA_WA1/2020, at a MOI of 0.01. After 72 h, the cells were collected from flasks, pelleted and fixed in 2.5% Glutaraldehyde (E.M. Sciences, Warrington, PA, USA), in Millonig’s sodium phosphate buffer (Tousimis Research, Rockville, MD, USA) for 72 h. After fixation was complete, the cells were washed repeatedly in Millonig’s buffer, and incubated for two hours in 1% osmium tetroxide at room temperature in the same buffer. Following rinsing steps in ultrapure water and en bloc staining with 2% uranyl acetate, the samples were dehydrated in a series of graded ethanol and infiltrated and embedded in Spurr’s plastic resin (E.M. Sciences, Warrington, PA, USA). Cured resin was cut to size and 80 nm thin sections were acquired at a microtome (Leica UC7, Leica Microsystems Inc., Buffalo Grove, IL, USA) for TEM quality check as well as to locate possible ROIs for FIB-SEM imaging. We observed viruses both inside cells, as well as in the extracellular space, in between adjacent cells. (Figure 1C, Figure 2, Supplementary Movie M1). In these samples, we noticed areas of relatively virus-free cells, and a few patches of cells with high counts of viral particles. In an approach developed in our group and independently reported recently [36], 5 µm thick sections were cut, collected on a substrate and carbon coated before imaging. For other aldehyde fixed samples and correlative experiments, we typically have used protocols following [74], and for high-pressure frozen samples, following [68].

### 4.3. Image Generation and Resolution

In FIB-SEM, BSE signals generate “TEM-like” images with inverted contrast. In return for efficient vEM imaging, the electron optics in FIB-SEM result in slightly lower image quality, especially apparent at higher magnifications (Figure 1C). In TEM and AT approaches, the sections can be interrogated at length, whereas in SBF-SEM and FIB-SEM, where imaging is coupled with sectioning or milling, there are other factors to consider in commercial setups:

1. Long dwell times (how long and how many times the electron beam visits each pixel) have diminishing returns. This adds to the imaging overhead, and as useful signal is typically extracted and noise averaged out within several µs, accumulated negative charges imparted to the sample result in “baking” of the resin and image degradation. A dwell time of 3–4 µs is usually sufficient.
2. At the parameters typically used (1–2 kV accelerating voltage, 0.5–2 nA current for the electron beam), beam widths are typically on the order of ~5 nm, meaning that artificial increasing of magnification ends up supersampling the ROI (pixel sampling << beam spot size) and blurring the image. Similarly, artificially setting very thin z slices risks including information from deeper within the sample, again causing image blurriness. Usually, either a ~5 nm z slice with faster imaging, or 10–20 nm slice with slower imaging is chosen.
3. FIBs have limited “sweep”: the FIB moves back and forth rapidly at the cliff face while inching forward to mill away controlled amounts of resin. A limit of ~ 100 µm width is practical, as milling meaningful depths at these widths necessarily slows down the FIB to the point that sample drift in the SEM chamber rivals the FIB advancement rate. It is possible to ablate away larger volumes, or alternatively fixed volumes faster, with the FIB operated at a higher current, but the user loses fine control with the larger beam profile. Further, unlike in the material sciences, soft and insulating resin polymers are prone to warping and uncontrolled sputtering at high FIB currents. Irrespective, FIB-SEM imaging is not a high-throughput approach: as a rule of thumb, ~10,000 µm^3^ or a volume of 25 × 20 × 20 µm per day is a reasonable limit without significantly compromising resolutions or signal-to-noise (SNR) ratios.
4. A simple empirical test for resolution is that at minimum, both membranes in the nuclear envelope and ER should be resolved and crisp in all planes throughout the run, and ideally, intercristal spaces should be visible in mitochondria. Automated and frequent beam tuning on the fly should prevent focus and stigmation issues, and these are immediately visible to the eye.

**Image acquisition in our experiments:** SEM imaging of the polished resin surface at 1.5 keV landing energy, 1 nA beam current and 10–20 nm pixel size revealed possible ROIs: cell-cell contacts with discernable viral particles (Figure 2A–D). After a brief confirmatory image, the ROI was protected by a patterned platinum (Pt) and carbon (C) pad of size 30 × 20 µm deposited by the FIB operated at 30 kV and 1.5 nA and revealed in orthogonal cross section by milling a trench at 30 nA. Note the 20 µm “y-axis length” of the Pt pad determines the maximum reliable milling distance (“z depth”) of a FIB-SEM image volume. After a polish mill at 1.5 nA, images were acquired at 3 or 4 nm pixel size and 3–4 µs total dwell time (1.5 keV/1 nA), with a FIB step size of 9 or 12 nm, respectively (30 keV/1.5 nA), in simultaneous mill-and-image mode. This resulted in a steady FIB advance rate into the resin, which in our hands should be 8 nm/min or above to reduce notch tracking instabilities. Beam tuning was performed automatically every 30 min and notch tracking after every slice. An imaging ROI of just under 30 × 6 µm (i.e., constrained by the pad edges in x and pad and substrate in y) was chosen, and a run of ~1000 images could be collected overnight (~16 h). This was sufficient to capture a significant portion, if not the entirety, of contacts in these relatively flat cells.

### 4.4. Processing and Correlation

Image processing of FIB-SEM or vEM datasets and correlation with other imaging modalities is an area of intense research and exciting developments [75,76]. There is a host of paid software products such as Amira (ThermoFisher Scientific, Waltham, MA, USA), Arivis (https://www.arivis.com/en/, accessed on 31 March 2021) and Imaris (https://imaris.oxinst.com/, accessed on 31 March 2021), hybrid products that allow free non-commercial use e.g., ORS Dragonfly (https://www.theobjects.com/dragonfly/index.html, accessed on 31 March 2021) as well as free, open source software and plugins therein, such as Fiji/ImageJ [77,78], 3Dslicer [79], MIB [80], IMOD [81], Ilastik [82], Vast [83], or solutions from manufacturers such as Apeer from Zeiss (https://www.apeer.com/home/, accessed on 31 March 2021) that allow registration, stitching, further processing, and segmentation. We urge newcomers to sample as many options as possible before deciding on one or several packages (and still look out for newer methods!); sub-optimal “quick fix” solutions may work temporarily but will waste time and resources or produce inaccurate results in the long run.

A FIB-SEM processing “pipeline” may involve the following steps, in no rigid sequence:

1. Registration and inversion: FIB-SEM images are typically acquired as grayscale.tiff files, with some drifts and jitters, and possibly poor SNR. 8-bit depth is sufficient for most applications and helps control file size. Contrast inversion is often an option during acquisition itself. A popular approach to register the newly acquired stack is to generate and apply transforms between successive images to correct for translation, rotation, skew etc. Some acquisition software can perform simple registration. The registration, stitching, and TrakEM2 [84] plugins in Fiji are popular, and some groups use custom solutions [85]. Stitching of FIB-SEM image tiles is unnecessary given small ROIs, but z-spacing correction [86] may be required.
2. Binning and Denoising: FIB-SEM imaging uniquely allows high-resolution 3-D reconstructions with isotropic voxels, enabling equal and warp-free sampling. Many groups directly acquire data with isotropic settings (e.g., 8 × 8 nm image pixel and 8 nm FIB step size), but it is also possible to acquire at say 4 × 4 × 8 and then “bin” or average by 2 in xy to yield final 8 nm voxel sizes. Binning averages out noise and reduces file size, but at the expense of pixel size, so this needs to be deployed wisely. A hidden advantage is that coarser z spacing speeds up FIB advancement rates, stabilizing milling and reducing artifacts. Various denoising algorithms may also be applied and most recently, deep learning-based approaches for denoising [87] are showing appreciable results.
3. Correlation: Combining FIB-SEM with other techniques, most frequently light or fluorescence microscopy (correlative light electron microscopy, CLEM) allows live, high-throughput screening or specific imaging of targets and 3-D ultrastructural reconstructions of the same regions of interest. Much work has been done recently, including an excellent handbook published on correlative imaging [75]. There are two connotations for CLEM: correlation for identification or relocation, where modalities like live light microscopy (LM) [66], cryo-LM [69], or XRM [88] are used to identify the coordinates to be imaged by FIB-SEM subsequently. The other is correlation for colocalization or registration, which requires high-resolution registration of signals from two modalities [89], which by default requires 3-D reconstructions for LM and FIB-SEM since the two imaging planes are orthogonal to each other. The correlation must take into account the differences in resolution, orientation, and differential warping and movements that occurs during sample processing. Recently developed software modules address these [90] but correlative experiments still need to be designed carefully to be correct and insightful.

**Image processing in our experiments:** Our FIB-SEM image stacks were aligned using custom scripts measuring cross correlation between subsequent images in a stack. The resulting transform file was applied to the raw images to yield a registered stack. This was contrast inverted, signal floated to a common range and binned in the imaging plane to yield a “clean” isotropic.mrc file used for reconstructions. For segmentation of coarse features, this file was cropped as required and the volume further binned by two in all axes to generate a small sub-volume at typically 16 nm voxel size. This file was usually <100 MB and could then be imported into segmentation and visualization software easily. Viruses could be visualized at this pixel sampling and were confirmed with the high-resolution image data.

### 4.5. Segmentation and Visualization

From a processed image stack or 3-D image volume, features of interest often need to be extracted or “segmented” for downstream visualization or analysis. The isotropic nature of FIB-SEM image volumes permits easy interactivity in all 3 planes or directly in 3-D; viewing and segmenting purely in the imaging plane (as a “z stack”) wastes this advantage and the resulting models suffer from a “stacked pancake” artifact. Many software packages natively allow 3-D functions, modeling and viewing (IMOD and 3DSlicer are good starting points). For groups lacking advanced computing resources, or that have one-off requirements, segmentation continues to be manual: slow, prone to biases, and largely non-transferable. Even when aided significantly by simple computational tools such as thresholding and filtering, the user may have to hand paint over recognizable features in a greyscale image dataset to create binary label maps that can be extracted. Crowd-sourced initiatives [91,92] can help alleviate some of the burden on individual labs, and VR-based viewing may help speed up proofreading [93], but the investigator needs to be realistic of the time needed to segment out features accurately and precisely from a FIB-SEM dataset, especially if the targets are numerous, large, or complex. Further, expert proof-reading is currently an essential step if manual segmentation has been outsourced.

In parallel, there have been two active areas of development: a number of software packages (Fiji, Ilastik, Dragonfly, others) now have native or add-on modules that allow computer-aided segmentation using either traditional or machine learning approaches, which, based on simple image-level concepts such as shapes, edges etc. can help generate a first pass segmentation that typically has to be cleaned up subsequently. The second area is in deep learning. There have been many recent examples where AI algorithms have successfully “learned” concepts that allow rapid and quite accurate segmentation of specific features [80,94,95,96], including solutions that exploit specifically the isotropic resolutions of FIB-SEM data [97], however these remain currently inaccessible to the non-aficionado for several reasons. Many of these “proof-of-principle” solutions do not transfer well to other questions or features of interest (for example, neural network models that recognize mitochondria in a vEM dataset of mouse brain currently fail to recognize mitochondria in say HeLa cells), although this is a point of intense work. Also, many solutions exist as scripts that take some know-how to execute, let alone understand, edit, improve, and apply to specific biological problems. This, however, is changing rapidly, and accessible tools from image analysts coupled with imaginative ways that the biologist approaches the question will hopefully soon help translate these advances to a wider audience.

Segmentation for our experiments: High school students working remotely were provided trimmed.mrc image volumes and 3DSlicer tutorials to generate segmented label maps for the plasma membranes, fiducials for viral particles and 3-D models. These were proof-read and edited by scientists using 3DSlicer or Dragonfly. Calculations of membrane curvatures on the mesh models were performed using Python scripts, and the heatmap LUT was applied in MeshLab.

## 5. Observations of SARS-CoV-2-Cell Interactions in 3-D

Here we show representative results from FIB-SEM imaging of SARS-CoV-2 virus-cell and cell-cell contacts in vitro. We have as of this writing been unsuccessful in observing virions from FIB-SEM data directly from patient autopsy samples, likely due to the difficulties in capturing these small particles in massive tissue volumes. We could not perform specific experiments (e.g., correlative LM/FIB-SEM) as we had limited access to samples. Instead, here we present examples of some qualitative and quantitative analyses possible by imaging such samples by FIB-SEM.

ROIs of cell-cell contacts with viral particles were either imaged by FIB-SEM in house (Vero E6 cells, Figure 2A–D, Supplementary movie M1) or downloaded from FIB-SEM data deposited on EMPIAR (Calu-3 cells, [15], Figure 2E–G). The ROIs were segmented aided by the watershed and threshold modules on 3DSlicer followed by manual clean up and rendering. These showed extensive contacts between adjacent cells (Figure 2A, brown and green) mediated by contiguous tight junctions (yellow, and Figure 2B) and some punctate focal connections (Figure 2C,D, purple). Interestingly, there is a dramatic surface viral density difference on either side of the tight junction (Figure 2C,D), suggesting that SARS-CoV-2 may “surf” [98] on plasma membranes of filopodia, and that this may be restricted by tight junctions. Experiments with more organized or 3-D epithelial layer cultures are warranted. Neighboring Calu-3 cells also had closely apposed plasma membranes (Figure 2E,F) with multiple desmosome-like contacts in the areas examined (Figure 2E,G); this appeared to have little impact in the spatial distribution of virus on either cell membrane (Figure 2E), presumably due to the lack of a contiguous barrier. The term “virological synapse”, akin to the immunological synapse, may be described as a cytoskeleton-dependent, stable adhesive junction across which virus is transmitted by directed transfer [99], and is understood to be relevant in the transmission of enveloped viruses between immune, epithelial, and other cell types [100,101,102]. It is tempting to term these cell-cell contacts as virological synapses; FIB-SEM imaging has been used to characterize these structures in cell-to-cell HIV transmission [63], and these have been documented by fluorescence microscopy in cell-to-cell transmission between coronavirus-infected cells [103], but we believe that in the absence of correlative evidence and a lack of clear polarization of organelles with concomitant concentration of virus in the contact region, this would be speculative. However, targeted CLEM experiments may clarify these intriguing observations in the future.

**Figure 3:**
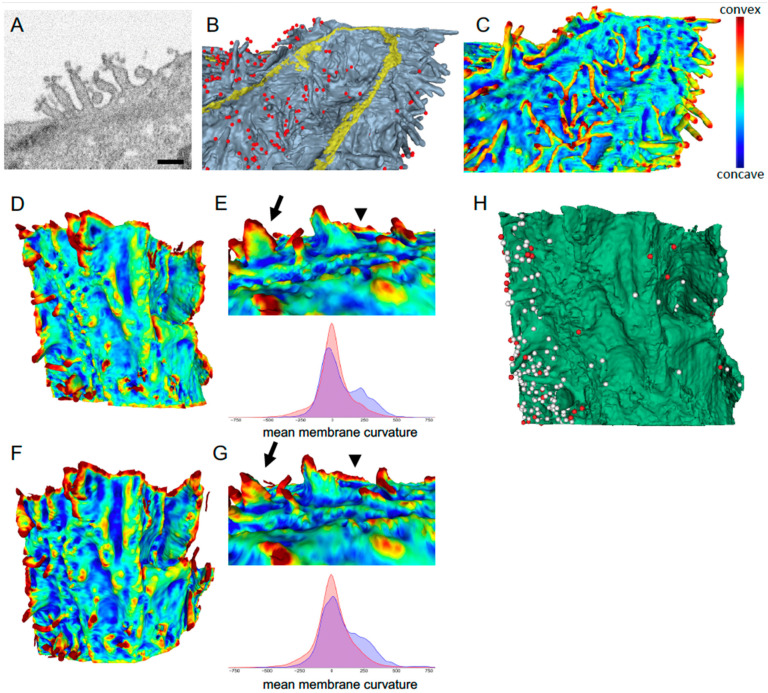
**Quantitation of membrane curvatures and viral densities from FIB-SEM**. (**A**) FIB-SEM micrograph showing viral particles preferentially associated with filopodia. Scale bar, 500 nm. (**B**) 3-D reconstruction of a SARS-CoV-2 infected Vero E6 cell. Gray, plasma membrane; yellow, tight junction; red, virus. (**C**) Heat map of the surface mean curvature of the cell in B. (**D**) Heat map of the surface mean curvature of the membrane depicted in Figure 2B,C (green cell) at 8 × 8 × 8 nm pixel sampling. (**E**) Top, heatmaps of representative sub-area, compare with (**G**). Bottom, correlation between a cell’s mean membrane curvature and the presence of virus particles from the FIB-SEM volume shown in (**D**). Blue, virus particles near the cell membrane (<100 nm distance). Red, randomly sampled points along the cell membrane. (**F**) Simulated SBF-SEM of the same surface in D, but now at 8 × 8 × 48 nm resolution. The sparser z sampling is reflected in horizontal ridges in the reconstruction. (**G**) Top, Heatmap corresponding to area in E. Arrow, warped features; arrowhead, sawtooth or ridge artifact in SBF-SEM but missing in FIB-SEM (**E**). Bottom, correlation between mean membrane curvature and presence of virus particles in (**F**,**H**). Same surface as (**D**) showing viral particles, white fiducials. Red fiducials, viral particles missed in the lower z resolution simulated SBF-SEM volume.

FIB-SEM imaging also allows quantitation of certain aspects of 3-D reconstructions. We highlight two that are relevant—plasma membrane curvatures and surface viral density, and we show how the high 3-D resolution of FIB-SEM is important to capture these features. Coronavirus particle egress is reported to occur by exocytosis of vesicles transported to the plasma membrane via the endoplasmic reticulum-Golgi intermediate compartment (ERGIC) secretory pathway [104,105]. In HIV-1 infection, infected cells use a variety of membrane protrusions, including those involved in the virological synapse, to infect target cells [61,106], and we have recently shown that HIV-1 virions preferentially bud from infected cell plasma membrane patches with positive curvatures as measured by FIB-SEM imaging [107]. Here, mean curvatures of smoothed models were calculated using the trimesh library in Python [108], and distances calculated radially from the centroids of fiducials manually placed atop viral particles. We saw that viruses were preferentially located at membrane patches with higher positive curvatures, such as the tips of filopodia, than the rest of the cell membrane (Figure 3A–C). This was evident by visual inspection of the curvature heat maps (Figure 3D) and importantly, this was also quantifiable and found to be significant (Figure 3E, bottom; average mean curvature of reconstructed Vero E6 plasma membrane ≤100 nm away from the closest viral particle as compared to >100 nm away from was 64.81 nm^−2^ and 7.96 nm^−2^, respectively; *n* = 249 virions, *p* < 0.0001).

Lastly, a measure of the importance of high-resolution imaging in all three axes was approximated by comparing a FIB-SEM segmented volume (Figure 3D) with a simulated SBF-SEM or AT volume (Figure 3F), where only every 6th slice of a FIB-SEM reconstruction was considered, i.e., 8 × 8 × 8 nm vs. 8 × 8 × 48 nm reconstructions. We saw that there were artifactual features and characteristic ridges in the 3-D model arising from the sparser z axis sampling, and these were visible even after smoothening (compare Figure 3E,G, top; arrows points to membrane structures altered in 3G, arrowheads point to ridges introduced in 3G). Further, there was a difference in the measured mean curvature associated with viral particles (blue curves in Figure 3E,G, bottom; 82.69 nm^−2^ for simulated SBF-SEM versus 64.81 nm^−2^ for FIB-SEM). Importantly, the sparser sampling resulted in 195 instead of 249 viral particles being counted confidently in the simulated SBF-SEM vs. FIB-SEM datasets (Figure 3H, missing virions in red), and even sparser sampling (say ~100 nm) starts introducing further artifacts (not shown). Thus, for experiments where small features such as SARS-CoV-2 viral particles need to be accurately accounted for and characterized architecturally, the unparalleled 3-D resolution of FIB-SEM can be an invaluable imaging tool.

### Data Handling and Sharing

The development of vEM techniques over the past decade has led to a well-documented “data tsunami” [109], creating new challenges for storage/retrieval, transfer, sharing and handling [110]. Data solutions at the point of acquisition, institution or in the cloud may or may not be relevant, fully developed, or accessible to the investigator paying for time at a core facility. Rather than prescribing specific solutions, we highlight several points for the scientist to consider, acknowledging the vastly different capabilities and conventions presently.

1. Raw vs. “clean” image data: Even with acquisition at 8-bit depth, raw image datasets on the order of TB are common these days; cropping, binning down and processing data to the minimum “clean” image volume required to answer the question at hand will greatly ease handling and transfers. One possibility is creating two data streams: one raw data to archive, and more volatile targeted sub-volumes which can be probed as required. Appropriate sub-volumes are sufficient for most correlation and segmentation, although care is needed to maintain spatial fidelity.
2. Derived data such as segmentation and quantitation: as the reporting of FIB-SEM data transitions from representative 2-D images to 3-D reconstructions to quantitative analyses from 3-D models, it is critical to track intermediate files and parameters used to ensure continuity of these derived data. Strong lab record practices and naming conventions help, and workflow builders such as KNIME can help teams repeat proven analysis pipelines or construct new ones. Further, as metadata conventions in vEM get established, converge, and finally integrated into imaging workflows, we expect that distinct data types will be easier to combine and connect.
3. Data sharing: While the advantages (and encouragement) of sharing data have become clear, we acknowledge a hurdle specific to FIB-SEM or vEM data. Often, only a small fraction of these datasets is mined and published, leading to some reluctance to share these large image volumes. We hope for more concrete incentives for data sharing in the future, but for now make the case for undeniable collaborative benefits (and gratification!) of such datasets being re-used for scientific progress. There are many institutional repositories such as EMBL-EBI is accepting vEM datasets via EMPIAR (https://www.ebi.ac.uk/pdbe/emdb/empiar/, accessed on 31 March 2021), with several large datasets already deposited.

Data sharing in our experiments: We generated data from CCL-81 (not shown) and Vero E6 cells in our laboratory. CCL-81 and Vero E6 cells are derived from kidney epithelium; both cell types express ACE-2 receptors and are valid models for SARS-CoV infection. However, we noticed that in a recent manuscript [15] the authors had performed an excellent FIB-SEM study of SARS-CoV-2 infected human lung epithelial cells. This dataset (EMPIAR-10490) was made available; we downloaded the data and used small representative sub-volumes to segment out viruses and plasma membranes (Figure 2E–G). This bolstered our confidence in our findings and provided intriguing parallels and differences that can now be separately investigated. We acknowledge Yannick Schwab and colleagues for uploading their data, and we do the same, see EMPIAR-10677 and Data Availability below.

## 6. Conclusions

Here we have attempted to provide the virologist or cell biologist with a practical primer to FIB-SEM imaging as a vEM approach as it pertains to capturing virus-cell interactions in 3-D and at high resolutions. Conventional TEM imaging is likely to continue to be regarded as the gold standard in virology; yet, it has the unmistakable limitation of being restricted to two dimensions and inseparable from manual “search and find” tradition. With vEM, an umbrella term for several similar but distinct approaches that includes FIB-SEM, the researcher sacrifices some resolution to varying degrees, and instead gains information in the third dimension across larger volumes. FIB-SEM is intrinsically amenable to automation, both during and after image acquisition. This creates its own set of issues as detailed above, but there is little doubt that FIB-SEM has the potential to reveal new biology in a variety of systems. One area to look forward to is the speeding up of FIB-SEM protocols in order to make it more practical in the diagnostic laboratory [111], and another is the application of ever more powerful deep learning and other computational approaches to extract meaningful insights from these giant datasets. At the same time, it behooves virologists to familiarize themselves with these methods and think imaginatively about research questions that fully exploit the potential of FIB-SEM, while being realistic and careful about their drawbacks. Much work is being done in this nascent field, and as more tools and technologies come into the fold, FIB-SEM and vEM are poised to make important impacts in virology in the future.

## Data Availability

FIB-SEM reconstructed image volumes, label maps and x y z locations of viral particles are available at EMPIAR-10677. https://www.ebi.ac.uk/pdbe/emdb/empiar/, accessed on 31 March 2021.

## Supplementary Materials

The following are available online at https://www.mdpi.com/article/10.3390/v13040611/s1. Movie M1: FIB-SEM volume and corresponding reconstructions of three Vero E6 cells infected with SARS-CoV-2. The three cells (blue, red, green) form tight junctions (yellow) with each other. Viral particles are shown as white spheres.
